# Immunoallergic Hepatitis Induced by Antitubercular Drug: A Case Report

**DOI:** 10.7759/cureus.78218

**Published:** 2025-01-29

**Authors:** Mohamed Lakhal, Meriem Rhazari, Sara Gartini, Afaf Thouil, Hatim Kouismi, Mohammed Aharmim, Jamal Eddine Bourkadi

**Affiliations:** 1 Department of Respiratory Diseases, Research and Medical Sciences Laboratory, Centre Hospitalier Universitaire Mohammed VI, Oujda, MAR; 2 Faculty of Medicine and Pharmacy, Mohammed I University, Oujda, MAR; 3 Department of Pulmonology and Phthisiology, Moulay Youssef Regional Hospital, Rabat, MAR; 4 Department of Respiratory Diseases, Ibn Sina University Hospital, Rabat, MAR

**Keywords:** antitubercular drugs, hypersensitivity to antitubercular drugs, immunoallergic hepatitis, pleuropulmonary tuberculosis, toxic hepatitis

## Abstract

Antitubercular treatment is associated with numerous adverse effects, among which immunoallergic reactions present a significant challenge in management. These reactions may sometimes lead to the discontinuation of one or more antitubercular drugs, potentially compromising the patient's recovery. We report the case of a 42-year-old patient, followed for pleuropulmonary tuberculosis, diagnosed clinically, and put on anti-bacillary treatment. The evolution was marked by the onset of hepatic cytolysis, revealed by vomiting that occurred five days later. The diagnosis of immuno-allergic hepatitis was based on the delay in the onset of symptoms and the rapid improvement in liver function after discontinuation of anti-bacillary treatment. After normalization of liver function tests, each drug was reintroduced progressively over three days, with close monitoring for cytolysis. Ethambutol, pyrazinamide, and isoniazid, in that order, were successfully introduced. Rifampicin desensitization was carried out and was uneventful.

## Introduction

Drug allergies are defined as pathological reactions induced by drug intake, mediated by an immunological mechanism [1]. Antitubercular treatment exposes patients to numerous adverse effects, among which immunoallergic reactions (IARs) represent a significant challenge in clinical management. These reactions can manifest in various forms, ranging from mild symptoms such as rashes or fever to more severe conditions such as hepatic involvement. Hepatic involvement in the context of an IAR is rarely described in the literature, making the positive diagnosis difficult to establish, which complicates their management and differential diagnosis. In some cases, these immunoallergic manifestations may require the discontinuation of one or more antitubercular drugs.

We describe, through the case of a patient presenting with a hepatic IAR, the clinical presentation, diagnostic approaches, and therapeutic management.

## Case presentation

A 42-year-old patient with no prior history of liver disease was being followed for pleuropulmonary tuberculosis, diagnosed clinically, and started on antituberculosis treatment based on rifampicin, isoniazid, ethambutol, and pyrazinamide. The clinical course was marked by the onset of hepatic cytolysis, clinically revealed by vomiting occurring five days after the initiation of treatment, in the context of fever.

Biologically, the alanine transaminase (ALT) level was 320 IU/L, nine times the upper limit of normal (<35 IU/L), while the rest of the hepatic function tests were unremarkable. A follow-up liver function test showed improvement after discontinuation of the anti-tuberculous treatment (ALT became 30 IU/L). In light of the observed hepatic cytolysis and as part of the etiological work-up, the patient’s history did not reveal any hepatotoxic drug use associated with the antitubercular treatment. The clinical examination did not show any cutaneous eruption (urticaria) suggesting an associated hypersensitivity reaction. There were no hematological abnormalities, particularly no eosinophilia, and the hepatic serologies were unremarkable. The liver ultrasound, performed to rule out a pre-existing liver disease, showed no abnormalities.

According to the literature, rifampicin is the most common drug associated with immuno-allergic reactions [2,3]. For this reason, and based on the data from our case (the early onset of symptoms five days after the initiation of anti-tubercular treatment and the rapid improvement in liver function tests after discontinuation of the anti-bacillary treatment), the diagnosis of immuno-allergic hepatitis secondary to rifampicin use was retained.

After the normalization of liver function tests, there was a progressive reintroduction of each of the other three drugs, which were less likely to cause this type of reaction, over three days, with close monitoring for any potential cytolysis. The introduction was successfully carried out in the following order: ethambutol, isoniazid, and pyrazinamide as shown in Table 1.

**Table 1:** Medication reintroduction protocol for our patient

Medication	First-day dose	Second-day dose	Third-day dose
Ethambutol	400 mg	800 mg	Full dose (1200 mg)
Isoniazid	50 mg	150 mg	Full dose (300 mg)
Pyrazinamide	400 mg	800 mg	Full dose (1600 mg)

As for the last drug, rifampicin, a desensitization procedure, both diagnostic and therapeutic, was performed according to the desensitization protocol developed by Matz et al. [4], and proceeded without incident.

## Discussion

Hypersensitivity to antitubercular drugs is one of the unpredictable adverse effects and can jeopardize the patient's life [2]. Hepatic involvement in the context of an immunoallergic reaction to antitubercular treatment is rarely reported in the literature. Antitubercular drugs can induce hypersensitivity reactions of types I to IV, according to the Gell and Coombs classification [3].

**Figure 1:**
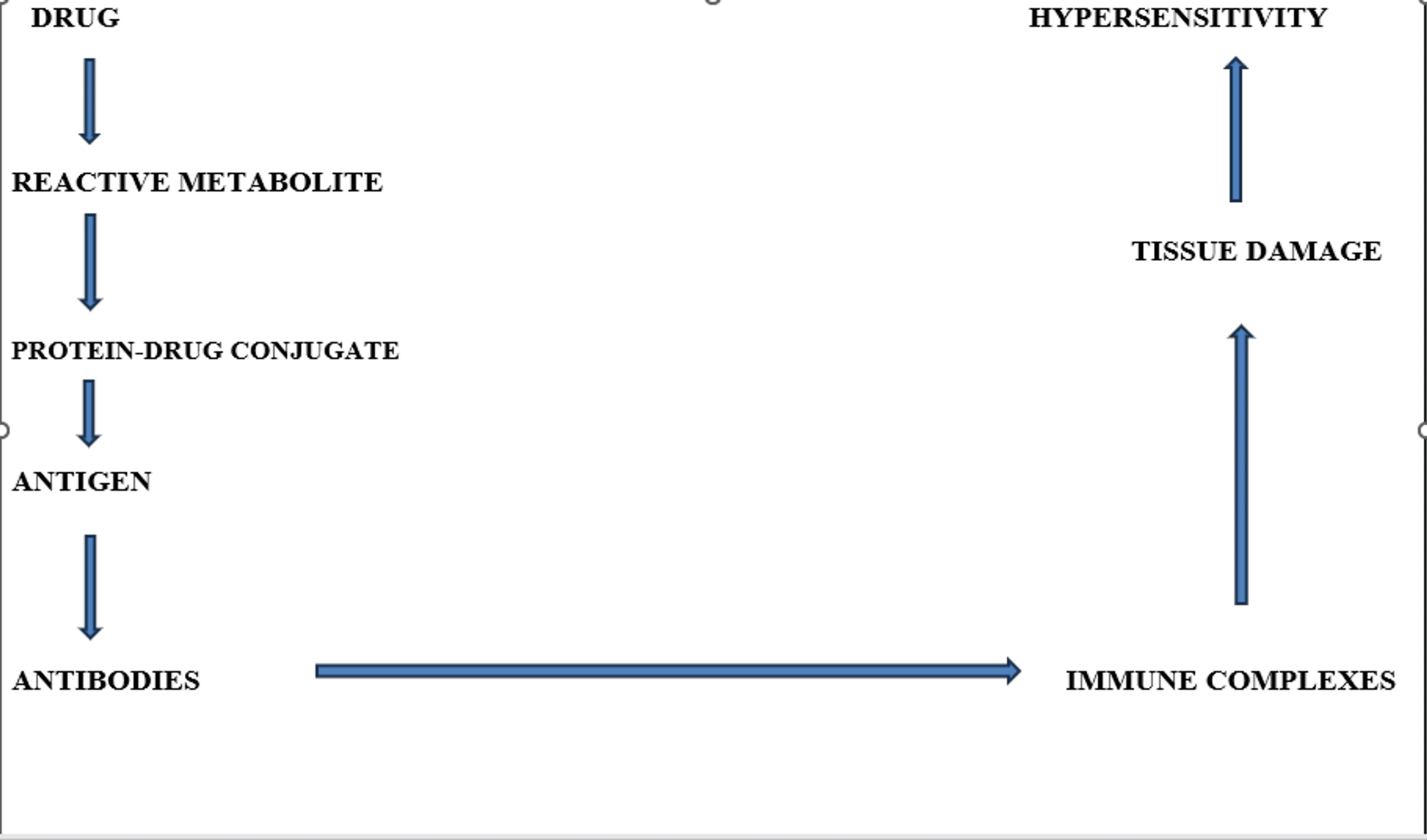
The mechanism of immunoallergic hepatitis: the formation of a reactive metabolite Image Credit: Authors

The mechanism of drug-induced hepatotoxicity of the immunoallergic type has led to numerous hypotheses and propositions. We will highlight the most commonly accepted, which involves the formation of a reactive metabolite [5]. Figure 1 illustrates the hapten formation hypothesis proposed by Park et al. [6].

Several stages lead to the appearance of immunoallergic hepatotoxic phenomena. The causative drug is first metabolized into a reactive metabolite. This unstable metabolite can then react with existing proteins and form a drug-protein conjugate. The protein, modified by the drug, becomes immunogenic. The immune system subsequently produces antibodies that form immune complexes with the antigenic protein. The final step involves tissue damage with hypersensitivity manifestations. This mechanism is a general process that can be adapted to the hepatic cell in this context [6]. The hepatocyte is the target of an immune reaction only if the involved antigen (protein modified by the drug) is expressed on the cell surface. This membrane expression may result from the migration of the antigen from the endoplasmic reticulum membrane, where the reactive metabolite is produced, to the cell membrane, or from the direct binding of the reactive metabolite to a membrane component. This protein-hapten structure then serves as the target for the immune system, leading to hypersensitivity manifestations [6].

Hepatic involvement could be immunoallergic or, in most cases, toxic. It does not have a specific clinical form and may present as cholestatic, cytolytic, or mixed. Rifampicin is the most commonly implicated antitubercular drug in these reactions [7]. The main differential diagnosis for immunoallergic hepatitis is toxic hepatitis. Generally, in a hepatotoxic reaction, there is a dose-dependent relationship with signs of toxicity. In contrast, in immunoallergic hepatitis, even a small dose can trigger a reaction. The onset of the reaction usually occurs after 15 days or more. Unlike immunoallergic hepatitis, clinical signs appear earlier in toxic hepatitis. The clinical signs of toxic hepatitis are usually mild. In contrast, in immunoallergic reactions, associated symptoms such as fever and a skin rash may be observed. Furthermore, improvement is slow in toxic reactions [8,9] (Table 2).

**Table 2:** Differentiating signs of toxic hepatitis from immunoallergic hepatitis

Signs Suggestive of Drug-Induced Toxic Hepatic Injury	Signs Suggestive of Drug-Induced Immunoallergic Hepatic Injury
Dose-effect relationship	No relation to dose
Onset of reaction: from 15 days onward	Less than 15 days
Clinical signs: poor	Associated extrahepatic manifestations: rash, fever, etc
Improvement: slow	Rapid resolution after discontinuation of the causative drug

In the case of a strong clinical suspicion of an immunoallergic reaction, the first step is the discontinuation of all medications. The second step involves identifying associated signs, particularly those indicative of an immunoallergic mechanism, such as fever and skin rashes. For medications that are less likely to be implicated, a gradual reintroduction over three days is carried out with close monitoring. For the most likely culprit drug, desensitization protocols are implemented. Desensitization, or tolerance induction, involves the gradual reintroduction of the drug responsible for the hypersensitivity reaction in order to force the development of tolerance to the drug. This process typically lasts from a few hours to several days, depending on the case and the protocol used. Its practice is not standardized and requires careful monitoring.

Several desensitization protocols have been described [4,10]; we used the protocol for rapid desensitization protocol developed by Matz et al. [4]. This protocol involves the gradual reintroduction of the drug, starting with a very low dose (0.1 mg), which is then doubled every 30 minutes until half of the target dose is reached on the first day. The full dose is administered on the second day. If a reaction occurs during administration, the drug will either be permanently discontinued if the reaction is severe, or the desensitization protocol will continue if the reaction can be managed with symptomatic treatment.

## Conclusions

Antitubercular drugs can trigger unpredictable, sudden, hypersensitivity reactions, posing diagnostic and therapeutic challenges. The diagnosis is made exclusively through a reintroduction test, which not only confirms the diagnosis but can also be therapeutic (most often) by allowing the patient to tolerate the medications. Close monitoring of patients on antitubercular treatment is essential to detect the onset of allergic reactions and ensure adherence to the treatment.
